# Glutathione S-Transferase pi-1 Knockdown Reduces Pancreatic Ductal Adenocarcinoma Growth by Activating Oxidative Stress Response Pathways

**DOI:** 10.3390/cancers12061501

**Published:** 2020-06-09

**Authors:** Rahul R. Singh, Jiyan Mohammad, Megan Orr, Katie M. Reindl

**Affiliations:** 1 Department of Biological Sciences, North Dakota State University, Fargo, ND 58108, USA; rahul.r.singh@ndsu.edu (R.R.S.); jiyan.mohammad@ndsu.edu (J.M.); 2 Department of Statistics, North Dakota State University, Fargo, ND 58108, USA; megan.orr@ndsu.edu

**Keywords:** GSTP1, pancreatic ductal adenocarcinoma (PDAC), oxidative-stress, JNK, ERK, Sp1, apoptosis, ROS, therapeutic targets

## Abstract

Glutathione S-transferase pi-1 (GSTP1) plays an important role in regulating oxidative stress by conjugating glutathione to electrophiles. GSTP1 is overexpressed in breast, colon, lung, and prostate tumors, where it contributes to tumor progression and drug resistance; however, the role of GSTP1 in pancreatic ductal adenocarcinoma (PDAC) is not well understood. Using shRNA, we knocked down GSTP1 expression in three different PDAC cell lines and determined the effect on cell proliferation, cell cycle progression, and reactive oxygen species (ROS) levels. Our results show GSTP1 knockdown reduces PDAC cell growth, prolongs the G_0_/G_1_ phase, and elevates ROS in PDAC cells. Furthermore, GSTP1 knockdown results in the increased phosphorylation of c-Jun N-terminal kinase (JNK) and c-Jun and the decreased phosphorylation of extracellular signal-regulated kinase (ERK), p65, the reduced expression of specificity protein 1 (Sp1), and the increased expression of apoptosis-promoting genes. The addition of the antioxidant glutathione restored cell viability and returned protein expression levels to those found in control cells. Collectively, these data support the working hypothesis that the loss of GSTP1 elevates oxidative stress, which alters mitogen-activated protein (MAP) kinases and NF-κB signaling, and induces apoptosis. In support of these in vitro data, nude mice bearing orthotopically implanted GSTP1-knockdown PDAC cells showed an impressive reduction in the size and weight of tumors compared to the controls. Additionally, we observed reduced levels of Ki-67 and increased expression of cleaved caspase-3 in GSTP1-knockdown tumors, suggesting GSTP1 knockdown impedes proliferation and upregulates apoptosis in PDAC cells. Together, these results indicate that GSTP1 plays a significant role in PDAC cell growth and provides support for the pursuit of GSTP1 inhibitors as therapeutic agents for PDAC.

## 1. Introduction

Pancreatic ductal adenocarcinoma (PDAC) is the third leading cause of cancer-related mortalities in the Western world and is responsible for more than 45,000 deaths per year in the US alone [1]. Less than 9% of PDAC patients survive for five years or more after diagnosis [2]. The conventional treatment approaches, such as chemotherapy, radiation therapy, surgery, and any combination of these, have had little impact on the course of this aggressive malignancy [3,4,5,6]. Therefore, new therapeutic strategies based on the unique molecular biology and physiology of pancreatic cancer are needed [7,8,9].

The constant need for cellular building blocks drives the overzealous metabolism in cancer cells [10]. As a result, abundant byproducts such as reactive oxygen species (ROS) and reactive nitrogen species persistently accumulate and dysregulate cellular homeostasis, causing DNA damage and inducing senescence [11,12]. To maintain optimal redox balance in the cells, efficient and counteractive antioxidant machinery is required. Glutathione (GSH), nicotinamide adenine dinucleotide phosphate (NADPH), and redox regulatory proteins such as thioredoxin reductase and thioredoxin constitute the antioxidant enzyme system and scavenge the high levels of ROS [13].

Glutathione S-transferase pi-1 (GSTP1) is a principal component of the antioxidant system [14]. It plays a cytoprotective role by catalyzing the conjugation reaction of reduced glutathione (GSH) to reactive electrophiles generated by cytochrome P450 metabolism [15]. GSTP1 is ubiquitously expressed in mammalian tissues and is overexpressed in human tumors of diverse anatomic origin [16,17], as well as in a wide variety of drug-resistant cell lines [18]. In addition to its role in cellular detoxification and glutathionylation, GSTP1 regulates stress-induced signaling by binding to and inhibiting the phosphorylation of c-Jun N-terminal kinase (JNK) [19]. Additionally, GSTP1 was recently shown to modulate glycolytic metabolism in breast cancer cells by enhancing glyceraldehyde-3-phosphate dehydrogenase (GAPDH) activity [20]. These, and additional literature [15,21,22,23], suggest that GSTP1 plays versatile roles in cancer cell survival, signaling mechanisms, and metabolism. With its established roles in breast [20] and cervical cancer [24], we postulate that overexpression of GSTP1 provides selective advantages to PDAC cells by scavenging elevated ROS and maintaining cellular homeostasis. In this present study, we provide evidence suggesting that GSTP1 contributes to pancreatic cancer cell growth and holds promise as a therapeutic target for PDAC.

## 2. Results

### 2.1. GSTP1 Is Overexpressed in Human PDAC Cells

**Figure 1:**
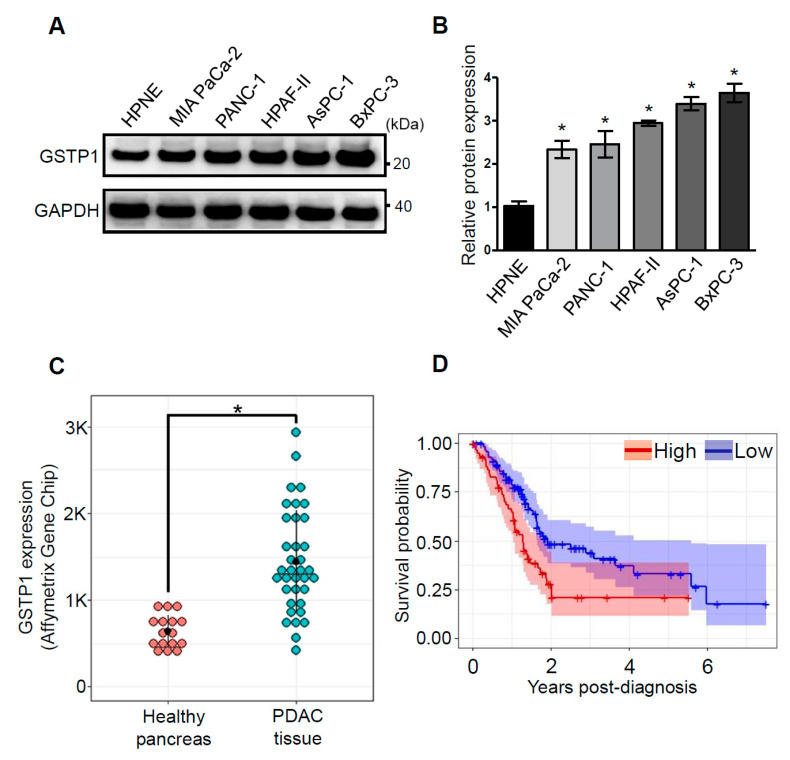
Glutathione S-transferase pi-1 (GSTP1) is overexpressed in human pancreatic ductal adenocarcinoma (PDAC) cells and tissues, and its expression is negatively correlated with patient survival. (**A**) GSTP1 expression in a normal pancreatic cell line (Human Pancreatic Nestin-Expressing ductal cells (hTERT-HPNE)) and a panel of human PDAC cell lines (MIA PaCa-2, PANC-1, HPAF-II, AsPC-1, and BxPC-3) was determined by Western blotting. Glyceraldehyde-3-phosphate dehydrogenase (GAPDH) protein levels were used as loading control. The images are representative of three independent experiments. (**B**) GSTP1 expression in MIA PaCa-2, PANC-1, HPAF-II, AsPC-1, and BxPC-3 cells were compared to GSTP1 expression in hTERT-HPNE cells. Densitometry values were determined using ImageJ software and normalized to GAPDH values. Student’s t-test was used to identify potential significant differences in expression in the tumor cell lines compared to hTERT-HPNE cells. Significant changes in GSTP1 protein expression are denoted with * (*p* < 0.05). (**C**) GSTP1 mRNA expression was compared in normal pancreas and PDAC tissue in the Gene Expression Omnibus (GEO) dataset submitted by Liewei Wang et al. (2009). Student’s t-test was used to analyze potential differences in GSTP1 mRNA expression for PDAC tissue compared to normal pancreas tissue. Significant changes in GSTP1 mRNA expression levels are denoted with * (*p* < 0.05). (**D**) The Human Protein Atlas was mined for GSTP1 mRNA expression in PDAC patients (*n* = 176) relative to their corresponding years of survival post-diagnosis. The cut-off value of 327 FPKM was used to divide patients in high- (red) and low- (blue) GSTP1-expressing groups. The Kaplan–Meier survival plot was constructed in RStudio. FPKM: fragments per kilobase of transcript per million mapped reads. Unprocessed images for the Western blotting results are shown in Figure S1.

GSTP1 is expressed at high levels in many human cancers, including colon, lung, breast, and ovarian cancers [25]. A higher expression of GSTP1 is correlated with disease progression and resistance to chemotherapeutic drugs [18]. However, the expression of GSTP1 is not well documented in human PDAC cells and tissues. We investigated the expression of GSTP1 in various PDAC cell lines. Intriguingly, we show that GSTP1 is present at higher levels in pancreatic carcinoma cell lines (MIA PaCa-2, PANC-1, HPAF-II, AsPC-1, and BxPC-3) compared to normal Human Pancreatic Nestin-Expressing ductal cells (hTERT-HPNE) (Figure 1A,B). Additionally, we compared the GSTP1 mRNA levels in human PDAC and healthy pancreas tissues in the publicly available Gene Expression Omnibus (GEO) dataset (GDS4102/200824_at). We found that GSTP1 is significantly overexpressed in PDAC tissue compared to the healthy pancreas (Figure 1C). Furthermore, using gene expression and survival data from The Human Protein Atlas [26], we determined that the overexpression of GSTP1 is negatively correlated with PDAC patient survival post-diagnosis (Figure 1D).

### 2.2. GSTP1 Knockdown Impairs PDAC Cell Growth

**Figure 2:**
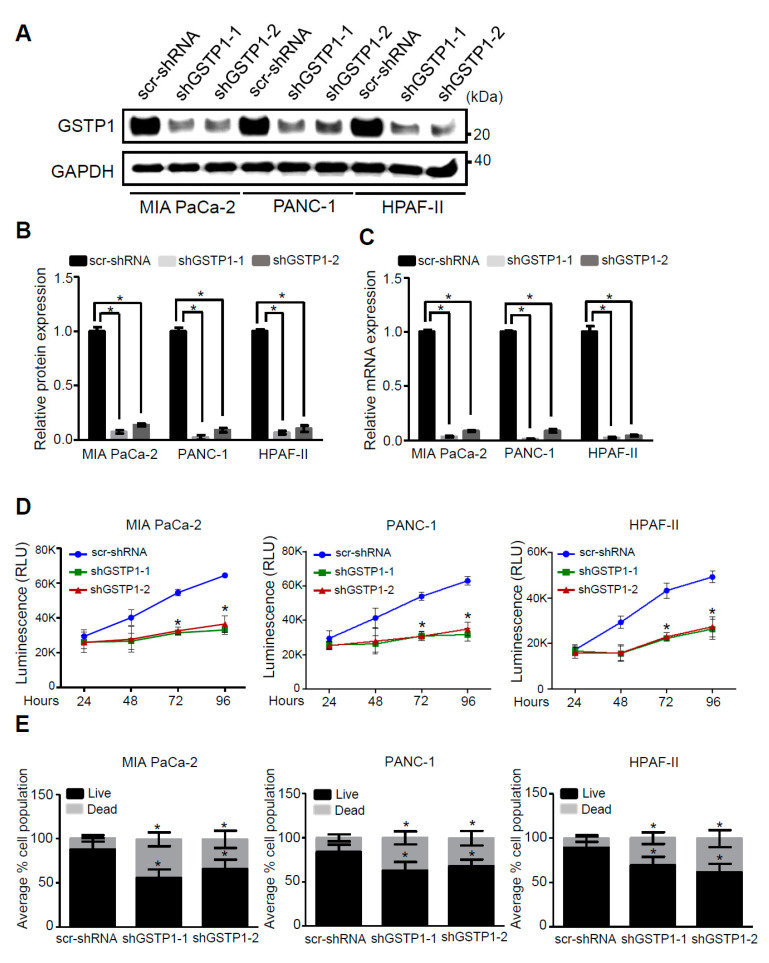
GSTP1 knockdown impairs PDAC cell viability. GSTP1 was knocked down in MIA PaCa-2, PANC-1, and HPAF-II PDAC cells using two independent shRNAs (shGSTP1-1 and shGSTP1-2) and expression was confirmed by (**A**,**B**) Western blotting and (**C**) quantitative real-time (qRT)-PCR analysis. Western blot data were normalized to GAPDH for each cell line, and relative protein expression is shown for the scrambled control shRNA (scr-shRNA) compared to the GSTP1 shRNA sequences. Protein and mRNA levels of GSTP1 in scr-shRNA were compared to shGSTP1-1 and shGSTP1-2. The images are representative of three independent experiments. Student’s t-test was used to evaluate the significance in the difference of GSTP1 expression among different groups. (**D**) CellTiter Glo^®^ assays were used to detect the average cell viability of control and GSTP1 knockdown MIA PaCa-2, PANC-1, and HPAF-II cells for two independent experiments with eight technical replicates for each. The y-axis represents the luminescence recorded after 24, 48, 72, and 96 h. The luminescence (cell viability) was compared between scr-shRNA and shGSTP1-1 and shGSTP1-2 independently. Student’s *t*-test was used to analyze the significance between knockdown groups and the control. RLU: relative luminescence units (**E**) 50,000 cells for control and GSTP1 knockdown MIA PaCa-2, PANC-1, and HPAF-II were seeded and the number of viable cells was counted using a trypan blue dye exclusion test after 72 h. The live and the dead cell populations for shGSTP1-1 and shGSTP1-2 were compared to the scr-shRNA. Student’s *t*-test was used to analyze for potentially significant differences. * denotes statistically significant differences between either GSTP1 knockdown and the control (*p* < 0.05). Unprocessed images for the Western blotting results are shown in Figure S1.

To elucidate the role of GSTP1 in PDAC cell survival, we developed two knockdown lines of GSTP1 (shGSTP1-1 and shGSTP1-2) in metabolically diverse human PDAC cells. MIA PaCa-2, PANC-1, and HPAF-II cells were transfected with GSTP1-specific shRNA and scrambled shRNA control plasmid (scr-shRNA) as described in the Materials and Methods section. MIA PaCa-2 and PANC-1 are mesenchymal in origin and lie towards the glycolytic end of the metabolic spectrum, while HPAF-II cells are epithelial and rely on lipolytic pathways for energy [27]. All these PDAC cells carry TP53 and KRAS mutations [28]. Following puromycin selection, the antibiotic-resistant cells were screened for GSTP1 knockdown by Western blot and quantitative real-time (qRT)-PCR analysis. Both shGSTP1-1 and GSTP1-2 resulted in more than a 95% reduction in GSTP1 protein expression (Figure 2A,B) and mRNA expression (Figure 2C) in all the three cell lines. To determine if GSTP1 knockdown can impair the viability of PDAC cells, we conducted CellTiter-Glo^®^ assays. We show that GSTP1 knockdown impairs cell viability for MIA PaCa-2, PANC-1 cells, and HPAF-II cells, by more than 50% for 72 and 96 h (Figure 2D). Similarly, trypan blue exclusion assays showed that GSTP1 knockdown increased the percentage of dead cells for all three PDAC cell lines by 25–30% compared to the control (Figure 2E). Supporting these results, we also show that GSTP1 knockdown reduces the clonogenic survival of PDAC cells (Figure S2).

### 2.3. GSTP1 Knockdown Elevates ROS Levels in PDAC Cells

**Figure 3:**
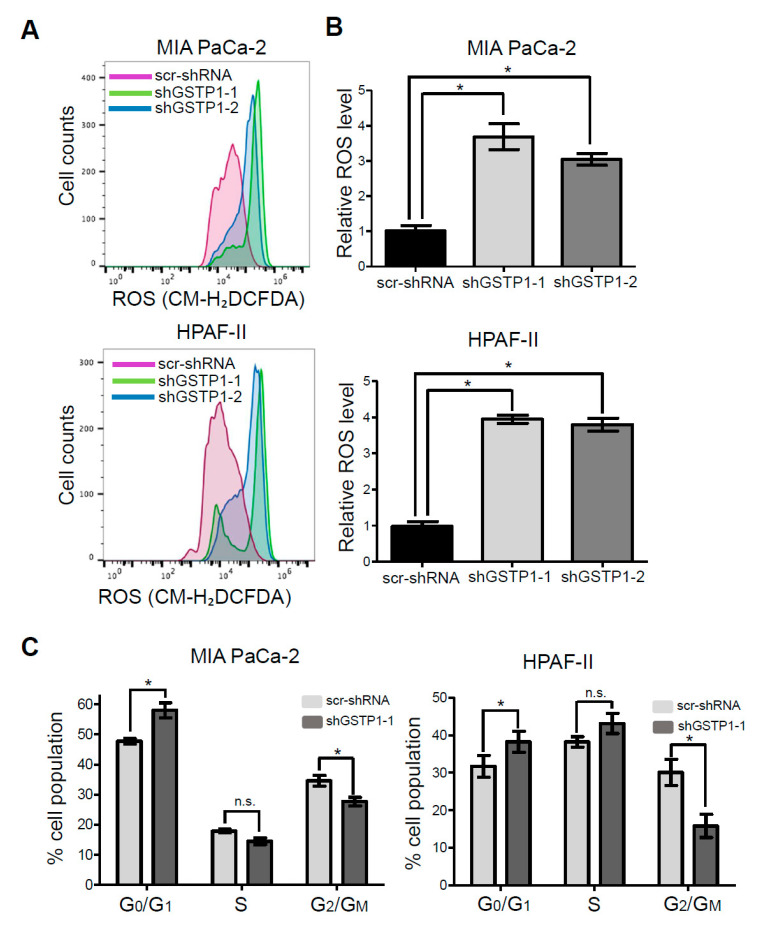
Effect of GSTP1 knockdown on the cell cycle profile and reactive oxygen levels (ROS) levels in PDAC cells. (**A**) Histograms showing ROS levels determined using CM-H_2_DCFDA and flow cytometry for control and GSTP1 knockdown MIA PaCa-2 and HPAF-II cells. The figure shows a representative image of three independent experiments. (**B**) uantification of ROS levels in control and GSTP1 knockdown MIA PaCa-2 and HPAF-II cells. ROS levels in scr-shRNA were compared to that in shGSTP1-1 and shGSTP1-2 independently. Student’s t-test was used to identify potential significant differences. (**C**) Control and GSTP1 knockdown MIA PaCa-2 and HPAF-II cells were analyzed for the percent cell population in different stages (G_0_/G_1_, S, and G_2_/M) of the cell cycle. The data shown represent the average percent cell population in the given phases of the cell cycle. The experiment was conducted three times for each cell line. The percentage cell populations in G_0_/G_1_, S, and G_2_/M phase of cell cycle were compared between scr-shRNA and shGSTP1-2. Student’s *t*-test was used to identify significant differences. * denotes statistically significant differences between GSTP1 knockdown groups and control (*p* < 0.05).

GSTP1, being a detoxification enzyme, has a key role in maintaining cellular homeostasis by scavenging reactive oxygen species (ROS) and protecting cells from oxidative damage [29]. We hypothesized that the growth inhibitory effects of knocking down GSTP1 result from the accumulation of ROS in PDAC cells. Control and GSTP1 knockdown (shGSTP1-1) MIA PaCa-2 and HPAF-II cells were stained with the fluorescent dye CM-H_2_DCFDA to detect ROS, and fluorescence was determined using flow cytometry. We show GSTP1 knockdown elevates ROS levels by at least three-fold in PDAC cells (Figure 3A,B).

### 2.4. GSTP1 Knockdown Prolongs the G_0_/G_1_ Phase of the Cell Cycle

Heightened ROS levels can activate transcription factors and several cell cycle regulatory proteins that inhibit the progression of cells through the cell cycle [21]. To elucidate the effects of GSTP1 knockdown on the cell cycle profile of PDAC cells, we identified the percentage of cells in each phase of the cell cycle via PI staining and flow cytometry. A larger percentage of GSTP1 knockdown PDAC cells were arrested in the G_0_/G_1_ phase compared to the control cells (Figure 3C). We found 57% of GSTP1 knockdown MIA PaCa-2 cells in G_0_/G_1_ phase compared to 47% of the control cells. Similarly, 38% GSTP1 knockdown HPAF-II cells were found in the G_0_/G_1_ phase compared to 31% of control cells. A complementary decrease in the G_2_/M population was observed in the GSTP1 knockdown PDAC cells. These results suggest that GSTP1 knockdown prevents PDAC cell proliferation by prolonging the G_0_/G_1_ phase.

### 2.5. GSTP1 Knockdown Activates Oxidative Stress-Mediated Apoptotic Signaling in PDAC Cells

**Figure 4:**
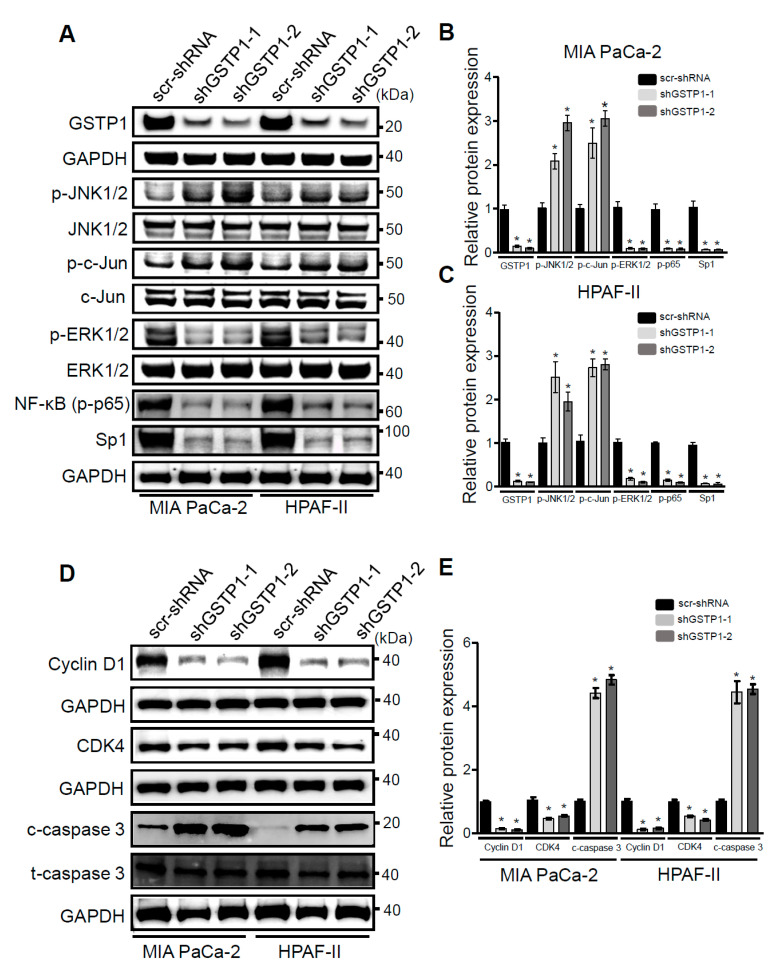

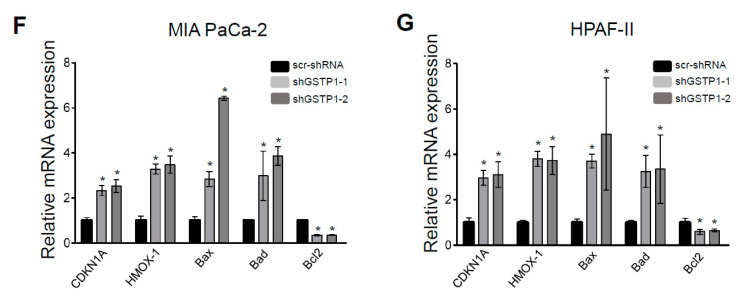
GSTP1 knockdown activates oxidative stress-mediated apoptotic and survival pathways in PDAC cells. (**A**) Phosphorylated (p-) levels of JNK1/2, c-Jun, extracellular signal-regulated kinase (ERK), and p65 and total specificity protein 1 (Sp1) protein expression were measured in GSTP1 knockdown PDAC cells via Western blotting. Levels of total JNK/2, c-Jun, and ERK1/2 were determined to confirm that changes in phosphorylated proteins were not due to changes in total protein level and to normalize the phosphorylated protein levels. GAPDH was used as a loading control. Changes in protein expression were quantified using densitometry for control and GSTP1 knockdown (**B**) MIA PaCa-2 and (**C**) HPAF-II cells. The protein levels were compared between shGSTP1-1 or shGSTP1-2 and scr-shRNA groups. The graphs show the ratio of phosphorylated proteins to total proteins in the knockdown groups relative to the scr-shRNA control. The figures show representative images for three independent experiments. (**D**) Protein levels of cyclin D1, CDK4 and activation (cleavage) of caspase-3 was analyzed in GSTP1 knockdown PDAC cells using immunoblotting. GAPDH and total caspase-3 were used as loading controls. (**E**) Cyclin D1, CDK4, and cleaved caspase-3 protein expression was quantified using densitometry. The figures show representative images for three independent experiments. The protein levels were compared between shGSTP1-1 or shGSTP1-2 and scr-shRNA groups. (**F**,**G**) Relative mRNA levels of *CDKN1A*, *HMOX-1*, *Bax*, *Bad*, and *Bcl2* were quantified using qRT-PCR in control (scr-shRNA) and GSTP1 knockdown (shGSTP1-1 and shGSTP1-2) for MIA PaCa-2 and HPAF-II cells. Student’s *t*-test was used to identify significant differences for the above experiments. Statistically significant changes in expression levels in GSTP1 knockdown groups compared to the control are shown with * (*p* < 0.05). Unprocessed images for the Western blotting results are shown in Figure S1.

GSTP1 has previously been reported to regulate the phosphorylation and activation of mitogen-activated protein (MAP) kinases [30]. GSTP1 inhibits the JNK signaling pathway by binding to JNK and preventing its phosphorylation. In response to oxidative stress, the JNK-GSTP1 complex dissociates [31], JNK is activated, and the downstream signal transduction leads to apoptosis [19,32]. Hence, we examined the effects of GSTP1 knockdown on the activation and phosphorylation of JNK1/2. We analyzed phosphorylated JNK1/2 protein expression through Western blotting. GSTP1 knockdown cells showed elevated phosphorylated JNK1/2 and its target protein, c-Jun, compared to the scrambled controls in the PDAC cells (Figure 4A–C).

To elucidate the role of GSTP1 in cell proliferation and cell survival, we also analyzed the expression of extracellular signal-regulated kinase (ERK1/2), the p65 subunit of NF-κB, and specificity protein 1 (Sp1) transcription factor in GSTP1 knockdown cells. GSTP1 knockdown cells had low levels of phosphorylated ERK1/2 and p65, and reduced Sp1 compared to the scrambled control MIA PaCa-2 and HPAF-II cells (Figure 4A–C). To explain the cell-cycle arrest phenotype of GSTP1 knockdown PDAC cells, we also investigated the expression of important cell cycle regulators. Interestingly, we found a large reduction in cyclin D1 protein expression and a moderate decrease in CDK4 protein expression (Figure 4D,E). We also found elevated mRNA expression of the cyclin and CDK complex inhibitor, *CDKN1A* (p21) (Figure 4F,G). Further, pro-apoptotic protein, cleaved caspase-3 (Figure 4D,E), and genes, *Bax* and *Bak* (Figure 4F,G), were up-regulated, while the anti-apoptotic gene *Bcl2* was downregulation in GSTP1 knockdown PDAC cells. *HMOX1*, an oxidative stress-associated gene, was also upregulated in GSTP1 knockdown PDAC cells (Figure 4F,G).

### 2.6. Addition of Glutathione Reverses the Effects of GSTP1 Knockdown on Cell Viability and Oxidative Stress-Response Signaling

**Figure 5:**
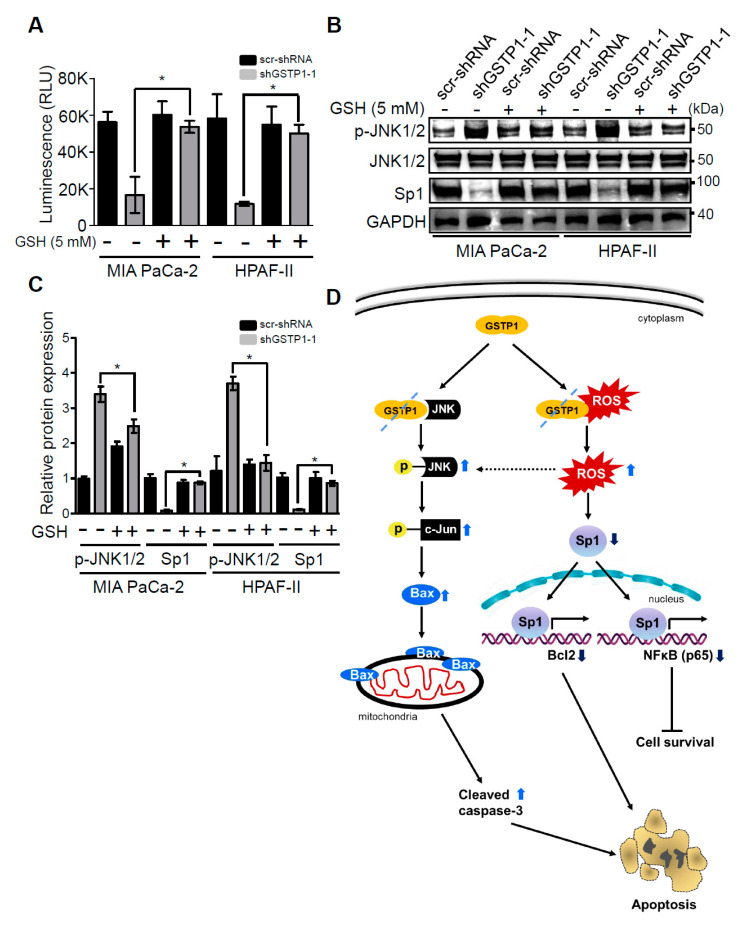
Exogenous antioxidant rescues the cytotoxic effects of GSTP1 knockdown in PDAC cells. Control and GSTP1 knockdown MIA PaCa-2 and HPAF-II cells were treated with 5 mM GSH for 72 h. (**A**) CellTiter-Glo^®^ assays were used to evaluate the average cell viability for three independent experiments with eight technical replicates for each. The luminescence (cell viability) was compared between shGSTP1-1 with and without GSH treatment. Student’s t-test was used to identify significant differences in growth in GSTP1 knockdown cells treated with or without GSH. RLU: relative luminescence units (**B**) Effects of GSH treatment on the phosphorylation of JNK1/2 and expression of Sp1 were determined using Western blotting. Total JNK and GAPDH were used as loading controls for the experiment. The figure shows one representative image of three independent experiments. Similar results were obtained in duplicate experiments. (**C**) Protein expression in two independent experiments was quantified using densitometry. p-JNK1/2 and Sp1 protein expression levels were compared between shGSTP1-1 with and without GSH treatment. Student’s t-test was used to identify the significant differences in protein expression in the GSTP1 knockdown cells treated with or without GSH. (**D**) Proposed mechanisms underlying the role of GSTP1 in pancreatic cancer cell proliferation, survival, and apoptosis based on Western blotting and qRT-PCR data. For all figures, * denotes statistically significant differences (*p* < 0.05). Unprocessed images for the Western blotting results are shown in Figure S1.

We next evaluated the ability of an exogenous antioxidant, glutathione (GSH), to reverse the cytotoxic effects of GSTP1 knockdown in PDAC cells. Control and GSTP1 knockdown MIA PaCa-2 and HPAF-II cells were treated with 5 mM GSH for 72 h. Our results show that GSH treatment attenuated the effects of GSTP1 knockdown in PDAC cells. The growth inhibitory effects of GSTP1 knockdown were significantly diminished upon GSH treatment (Figure 5A), suggesting that the accumulation of endogenous ROS is a leading cause of reduced cell survival in GSTP1 knockdown cells. We not only see the reduced expression of p-JNK in GSH-supplemented GSTP1 knockdown cells, but also the protein expression of Sp1 was found to be restored (Figure 5B,C). Overall, these results indicate that the loss of GSTP1 function surges ROS levels, activates JNK, and suppresses Sp1, which leads to changes in gene expression associated with oxidative stress, cell proliferation, survival, and cell death (Figure 5D).

### 2.7. GSTP1 Knockdown Impairs the Growth of Orthotopic PDAC Tumors In Vivo

**Figure 6:**
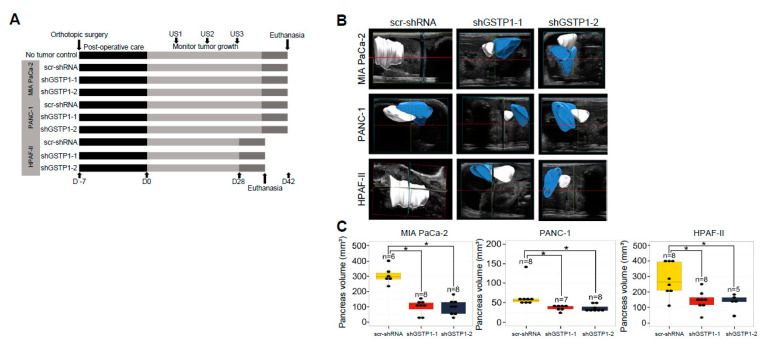

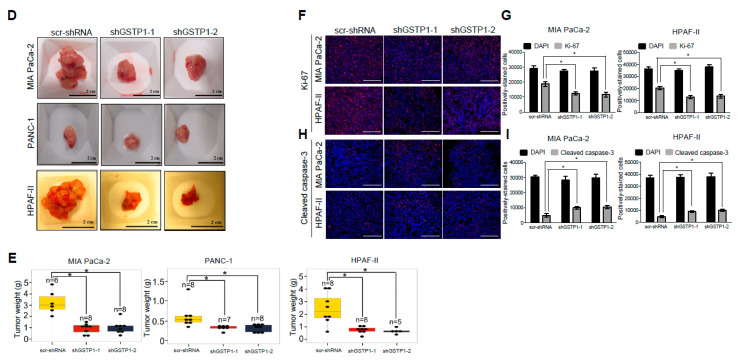
GSTP1 knockdown impedes the growth and proliferation of PDAC cells in vivo. (**A**) Schematic representation of animal experiments to assess the effects of GSTP1 knockdown on PDAC (MIA PaCa-2, PANC-1, or HPAF-II) tumor growth in nude mice for up to 42 days (D42). US1-3: ultrasound imaging was performed every 10 days to monitor tumor growth. For the HPAF-II group, the last ultrasound was done on D27 rather than D30. (**B**) Pancreatic tumor development was monitored and imaged using the FUJIFILM Vevo3100 ultrasound imaging system. The data show the tumor volume for one representative mouse for each group measured at US3 soon before euthanasia. Blue: healthy pancreatic tissue, white: pancreatic tumor tissue. (**C**) Total pancreata volumes were calculated using 3-dimensional ultrasound images for each cell line using the data collected at US3. (**D**) Size and (**E**) weight of the pancreata are shown for control and GSTP1 knockdown tumors. The figures show representative images of the tumor volumes (ultrasound) and tumor sizes of various treatment groups. The tumor volume and weight were compared between scr-shRNA and shGSTP1-1 and shGSTP1-2 independently. Welch’s one-way analysis of variants was performed to analyze the significant differences in tumor volume and weight between knockdown groups and the control. Tumor tissue sections from GSTP1 knockdown and scrambled controls for MIA PaCa-2 and HPAF-II were subjected to immunohistochemistry. (**F**) Ki-67 staining for control and GSTP1 knockdown MIA PaCa-2 and HPAF-II tumors. Scale bar: 200 μm (**G**) The quantification of the Ki-67-positive cell population. (**H**) Cleaved caspase-3 staining for control and GSTP1 knockdown MIA PaCa-2 and HPAF-II tumors. Scale bar: 200 μm (**I**) The quantification of cleaved caspase-3-positive cells. One representative image for each treatment group is shown. The percentage of Ki-67- and cleaved caspase-3-positive cells was determined by normalizing the number of Ki-67- and cleaved caspase-3-positive cells to that of 4′,6-diamidino-2-phenylindole (DAPI)-stained cells. Each value in the graph is the mean ± SD from 5-6 mice from each treatment group. * denotes statistically significant differences for all graphs (*p* < 0.05).

Intrigued by the in vitro growth inhibitory effects of GSTP1 knockdown, we next explored these effects in an orthotopic animal model of PDAC. Control and GSTP1 knockdown MIA PaCa-2, PANC-1, and HPAF-II cells were orthotopically transplanted into the pancreata of nude mice (Figure 6A). The tumor volume was monitored every ten days using FUJIFILM Vevo3100 ultrasound imaging system and was compared among the control and the GSTP1 knockdown groups. At the conclusion of the experiment, we observed decreased tumor growth via abdominal ultrasounds in GSTP1 knockdown groups compared to the controls (Figure 6B,C). Furthermore, our results show that GSTP1 knockdown PDAC cells generated significantly smaller tumors (*p* < 0.05) with a 50–80% reduction in tumor weight compared to the control (Figure 6D,E).

### 2.8. Tumor Cell Proliferation Is Reduced and Apoptosis Is Increased by GSTP1 Knockdown in Pancreatic Tumors

To explain the dramatic reduction in tumor size in GSTP1 knockdown cells, we evaluated the expression of the nuclear proliferation marker Ki-67 and the apoptotic marker cleaved caspase-3 by immunohistochemistry in mouse tumor tissues. The scrambled controls from the two PDAC cell lines showed 64% and 67% Ki-67-positive cells (Figure 6F). In comparison, tumors obtained from GSTP1 knockdown cells showed a notable reduction in Ki-67 expression for MIA PaCa-2 (37% and 35% for shGSTP1-1 and shGSTP1-2, respectively) and HPAF-II (38% and 29% for shGSTP1-1 and shGSTP1-2, respectively) (Figure 6G). Additionally, tumors obtained from GSTP1 knockdown cells showed an impressive increase in the expression of cleaved caspase-3 compared to the scrambled controls (Figure 6H,I). These data provide additional affirmation that GSTP1 knockdown impedes proliferation and promotes cell death via apoptosis in PDAC cells in vivo.

## 3. Discussion

Our data provide convincing evidence that GSTP1 plays a critical role in regulating PDAC cell growth, which was previously unknown. In this study, we show that the GSTP1 knockdown impairs the growth and proliferation of PDAC cells in vitro. We show, for the first time, that GSTP1 inhibition is associated with enhanced JNK activity and suppressed ERK, NF-κB, and Sp1 activity in PDAC cells. Furthermore, in an orthotopic pancreatic cancer mouse model, GSTP1 knockdown tumors showed an impressive reduction in growth compared to control tumors. Together, our results indicate that GSTP1 inhibition impairs PDAC cell growth, suggesting that GSTP1 is a viable target for PDAC therapy.

The ubiquitous expression of GSTP1 in a wide array of tissues and organisms provides evidence that GSTP1 has important cellular roles. We found GSTP1 protein expression was at least two times higher in five PDAC cell lines compared to normal pancreatic epithelial cells. Similarly, GSTP1 mRNA was reported in high levels in human PDAC tissue compared to the healthy pancreas tissue. Our analysis of The Human Protein Atlas [26] data revealed that elevated GSTP1 expression is associated with poor survival of PDAC patients, post-diagnosis of the disease. Previous research has shown that GSTP1 is expressed at high levels in a variety of human cancers, including colon, lung, breast, and ovarian cancers [25]. GSTP1 overexpression is associated with resistance to chemotherapeutic drugs like cisplatin, carboplatin, adriamycin, and bleomycin in ovarian and cervical cancer [33].

GSTP1 is associated with a variety of cellular processes, including detoxification [15], glutathionylation [21], actin polymerization [22], nitric oxide signaling [23], kinase signaling [31], and cellular metabolism [20]. To investigate the role of GSTP1 in PDAC cells, we generated two GSTP1 knockdown lines for each of the three metabolically diverse PDAC cell lines (MIA PaCa-2, PANC-1, and HPAF-II). MIA PaCa-2 and PANC-1 cells are poorly differentiated mesenchymal-type PDAC cell lines compared to HPAF-II cells that belong to an epithelial subtype [27]. GSTP1 knockdown significantly impaired the in vitro viability of all three PDAC cell lines, suggesting that this protein is vital to PDAC growth regardless of metabolic subtype. Our cell viability data are in concordance with previous reports where Louie et al. [20] described that GSTP1 knockdown impairs the growth of triple-negative breast cancer cells. They concluded by demonstrating that GSTP1 inhibition disrupts glycolytic metabolism, resulting in reduced levels of lipids, nucleotides, and ATP. Furthermore, recently, Fujitani et al. [34] showed that knocking down GSTP1 in cancer cells of various anatomic origins gives rise to mitochondrial stress and severely impairs cell proliferation. Interestingly, they found that pancreatic cancer cell growth was particularly sensitive to GSTP1 knockdown.

Attempts have been made to disrupt the cellular redox balance through pharmacological regulation in favor of increasing intracellular ROS and inducing apoptosis for the treatment of cancer. Arrick et al. [35,36] showed that specifically inhibiting the synthesis of GSH contributed to the destruction of neoplastic cells in vitro. Inhibiting GSTP1, an integral component of the cellular antioxidant system, is one avenue to disrupt redox balance. GSTP1 protects cells from electrophiles that cause oxidative damage to DNA, proteins, and lipids by conjugating electrophiles to GSH [18]. Here, we observed that the knockdown of GSTP1 in PDAC cells resulted in elevated ROS levels. Furthermore, the addition of GSH to GSTP1 knockdown cells enhanced cell viability and reduced the expression of stress and apoptosis-associated proteins. A previous study showed that an antioxidant (N-acetylcysteine) could reduce ROS levels in GSTP1 knockdown PDAC cells [34]. We speculate that GSTP1 knockdown impairs the ROS scavenging function that leads to ROS accumulation in PDAC cells. Our observations complement a previous report that showed GSTP1 inhibition using siRNAs and a pharmacological inhibitor elevated ROS levels and caused DNA damage in prostate cancer cells [29]. Moreover, GSH also restored cell viability, reduced ROS, and decreased apoptosis-associated protein expression in PDAC cells treated with a GSTP1 inhibitor [37].

Elevated oxidative stress activates the JNK signaling pathway and triggers apoptosis [31]. In a non-stressed environment, GSTP1 binds and inhibits the phosphorylation of JNK preventing the transcriptional activation of downstream cell stress pathways. However, under oxidative stress conditions, GSTP1 dimerizes into aggregates and its binding to JNK is deterred, enabling JNK activation [38]. Previously, we showed that the interaction between JNK and GSTP1 is interrupted in PDAC cells treated with a GSTP1 inhibitor [39]. Additionally, complementing our current results, it was shown that a JNK inhibitor could restore viability of PDAC cells treated with a GSTP1 inhibitor [39]. As expected, GSTP1 knockdown increased the expression of phosphorylated JNK and its downstream target, c-Jun, in PDAC cells. This increase could be due to elevated levels of ROS that could activate JNK signaling and/or reduced levels of GSTP1 that would also result in enhanced JNK signaling. Our data are supported by a previous report that suggested GSTP1 knockdown elevated phosphorylated JNK expression in cervical cancer cells [24]. The extent and duration of JNK activation can lead to ER stress, mitotic arrest, and eventually apoptosis in cancer cells [31].

To elucidate additional mechanisms through which GSTP1 knockdown impedes growth and the proliferation of PDAC cells, we investigated the activation status of ERK, NF-κB, and Sp1 pathways. GSTP1 knockdown cells displayed reduced phospho-ERK and NF-κB, and reduced Sp1 protein expression. In support of this, ERK and NF-κB protein expression were reduced in cervical cancer cells upon GSTP1 inhibition [24]. Sp transcription factors are upregulated in various cancer cells [40] and act as negative-prognostic markers for patient survival [41]. Our data are supported by previous reports that suggest ROS induction by chemotherapy and other anti-cancer agents lead to the downregulation of Sp proteins [42,43,44,45] and the reduced phosphorylation of ERK1/2 [46]. Similar to the previous studies [40,47], we show that the restoration of Sp1 expression can be achieved by supplementing the cells with an exogenous antioxidant such as glutathione. Additionally, Sp (1, 3, or 4) knockdown induced similar cellular responses, such as enhanced cell death, and gene expression changes (increased apoptosis promoters and decreased apoptosis inhibitors) as we observed in GSTP1 knockdown PDAC cells [48]. Furthermore, we show the reduced expression of principal cell-cycle regulators, cyclin D1 [49] and CDK4 [50,51]. Based on these results, we propose a mechanism through which GSTP1 alters MAP kinases and NF-κB signaling, averts apoptosis, and promotes cell survival and proliferation (Figure 5D). We speculate that in the absence of GSTP1, JNK is freely phosphorylated as a result of activating the downstream cell death pathways. Moreover, elevated ROS levels reduce the expression of Sp1 that transcribes *Bcl2* [52,53] and the p65 subunit of NF-κB [52,54]. Reduced levels of *Bcl2* and p65 in GSTP1 knockdown PDAC cells contribute to the apoptotic phenotype and decreased cell survival, respectively.

Intriguingly, we found that the orthotopic implantation of GSTP1 knockdown cells in the pancreata of athymic nude mice resulted in drastically smaller tumors compared to scrambled controls in terms of both tumor weight and volume. We also observed a lower percentage of proliferating cells and a larger population of apoptotic cells in tumors generated from GSTP1 knockdown PDAC cells. These results support our in vitro data as well as previously published literature. shRNAs targeting GSTP1 were shown to reduce breast cancer xenograft implants by more than three-fold [20]. Similar results were observed when GSTP1 was inhibited using specific morpholinos in cervical cancer [24].

Given GSTP1’s cytoprotective roles in xenobiotic detoxification, chemotherapeutics, and modulating oxidative stress, GSTP1 inhibitors emerged as promising anti-cancer compounds [55,56] and have been used alone or in combination with chemotherapeutic drugs [57]. The selective targeting of GSTP1 using 6-(7-nitro-2,1,3-benzoxadiazol-4-ylthio)hexanol (NBDHEX) has shown increased efficiency of chemotherapeutic drugs in melanoma [56]. A potent GSTP1 inhibitor, TLK199 (Telik Inc.), has been shown to modulate cell proliferation in human myeloid leukemic cells [58] and is under clinical trial for myelodysplastic syndrome [59]. LAS17 was recently developed as a highly potent and selective GSTP1 inhibitor that impairs breast cancer pathogenicity [20]. The aforementioned GSTP1 inhibitors have shown effective impairment in GSTP1 activity; however, their toxicity in normal cells is not well characterized.

Collectively, our findings illustrate the crucial role of GSTP1 in the growth of PDAC cells. The loss of GSTP1 function leads to the activation of oxidative-stress response pathways that trigger a cell death mechanism. Taken together, our data suggest that GSTP1 is a potential and promising novel therapeutic target to treat pancreatic cancer patients.

## 4. Materials and Methods

### 4.1. Chemicals

Puromycin was purchased from Sigma-Aldrich, St. Louis, MO, USA. A CellTiter-Glo^®^ luminescent cell viability assay kit was purchased from Promega, Madison, WI, USA. Ki67 antibody was purchased from Vector Labs, Burlingame, CA, USA. GSTP1 antibody was obtained from Santa Cruz Biotechnology, Dallas, TX, USA. Antibodies to GAPDH, β-actin, phospho-JNK (Thr 183/Tyr 185), total JNK, p65, pERK, total ERK, cleaved caspase-3, total caspase-3, phospho-c-Jun (Ser 73), total c-Jun, and Sp1 were obtained from Cell Signaling Technology, Danvers, MA, USA. Horseradish peroxidase (HRP)-linked anti-mouse and anti-rabbit IgG secondary antibodies were obtained from Cell Signaling Technology. CF633-conjugated goat anti-mouse IgG secondary antibody was obtained from Biotium, Fremont, CA, USA. CM-H_2_DCFDA was purchased from Life Technologies, Carlsbad, CA, USA. Glutathione was purchased from Calbiochem, Burlington, MA, USA.

### 4.2. Cell Culture

Human PDAC cell lines (MIA PaCa-2, PANC-1, HPAF-II, AsPC-1, and Bx PC-3) were obtained from American Type Culture Collection, Manassas, VA, USA. hTERT-HPNE cells were obtained from Dr. Channing Der’s laboratory at UNC, Chapel Hill, NC. MIA PaCa-2 cells were cultured in DMEM (Dulbecco’s Modified Eagle Medium) high-glucose media (GE Healthcare Life Sciences, Chicago, IL, USA) containing 10% (*v/v*) fetal bovine serum (Atlanta Biologicals, Flowery Branch, GA, USA) and 2.5% (*v/v*) horse serum (Corning, Corning, NY, USA). PANC-1 cells were cultured in DMEM high-glucose media containing 10% (*v/v*) FBS. HPAF-II cells were cultured in Eagle’s Minimum Essential Medium (EMEM) (Corning, Corning, NY, USA) containing 10% *v/v* Fetal Bovine Serum (FBS). AsPC-1 were cultured in RPMI-1640 (GE Healthcare Life Sciences) containing 10% FBS (*v/v*). Cells were maintained at 37 °C with 5% CO_2_. The cell lines were subcultured by enzymatic digestion with 0.25% trypsin/1 mM EDTA solution (GE Healthcare Life Sciences, Chicago, IL, USA) when they were 80% confluent. All cell lines tested negative for *Mycoplasma* contamination.

### 4.3. Constructing Knockdown Cell Lines

We used two independent short-hairpin oligonucleotides to knock down the expression of GSTP1. Lentiviral particles containing the shRNA (Sigma, St. Louis, MO, USA, catalogue# SHCLNV-NM_000852) were used to infect the target PDAC cell lines with polybrene (Sigma-Aldrich, St. Louis, MO, USA). Transfected cells were selected over five days with 5 μg/mL puromycin. The short-hairpin sequences used to achieve the knockdown of GSTP1 expression were: shGSTP1-1, CCGGCCTCACCCTGTACCAGTCCAACTCGAGTTGGACTGGTACAGGGTGAGGTTTTG; shGSTP1-2, CCGGC GCTGACTACAACCTGCTGGACTCGAGTCCAGCAGGTTGTAGTCAGCGTTTTTG. Scrambled GSTP1 shRNA, empty vector (pLKO.1), and shRNA targeting GFP were used as controls. Knockdown was confirmed by qRT-PCR and Western blotting techniques.

### 4.4. Western Blotting

Cells and tissues were lysed in lysis buffer (Promega, Madison, WI, USA) containing both protease and phosphatase inhibitors. Denatured proteins were resolved on 11% SDS-polyacrylamide gel and transferred to nitrocellulose membrane (Amersham^TM^ Protran^TM^ 0.2 μM, GE Healthcare Life Sciences, Chicago, IL, USA) using the wet electroblotting system (BioRad, Hercules, CA, USA). Blots were blocked using 5% BSA in Tris-buffered saline containing Tween 20 (TBS-T) solution for 1 h at room temperature, washed in TBS-T, and probed with primary antibody overnight at 4 °C. Following washes with TBS-T, the blots were incubated with HRP-linked secondary antibody at room temperature for 1 h. Blots were treated with SuperSignal West Femto Maximum Sensitivity Substrate (Thermo Scientific, Waltham, MA, USA) and visualized using FluorChem FC2 imaging system. The expression levels were quantified using ImageJ software. The data represent average ± standard deviation for three independent experiments.

### 4.5. RNA Extraction and Gene Expression by qRT-PCR

Total RNA was extracted using SurePrep TrueTotal RNA purification kit (Carlsbad, CA, USA) following the manufacturer’s instructions. cDNA was synthesized using 500 ng of total RNA and the qScript cDNA synthesis kit (Quanta Biosciences, Beverly, MA, USA). Steady-state RNA levels were determined as described elsewhere [60]. The relative change in gene expression was calculated using the 2−ΔΔCt method [61]. HPRT, β-actin, and β-tubulin were used as internal controls. The data represent the average ± standard deviation for three independent experiments with two technical replicates each. The primer sequences of the genes analyzed are listed in Table 1.

### 4.6. Cell Viability Assay

MIA PaCa-2, PANC-1, and HPAF-II cells (3000/well) were seeded in 96-well plates. The viability of control and GSTP1 knockdown cells after 24, 48, 72, and 96 h was evaluated by adding 100 μL of CellTiter-Glo^®^ substrate to each well containing 100 μL of media. The plates were incubated for ten min at room temperature. The endpoint luminescence was measured using Synergy H1 Hybrid multi-mode plate reader (Winooski, VT, USA) located in the Core Biology Facility, Chemistry and Molecular Biology, North Dakota State University. The gain was maintained at 135 and the integration time of 1 s using the Gen5 v2.07 software. The data represent the average ± standard deviation of three independent experiments with eight technical replicates for each treatment.

### 4.7. Cell Cycle Arrest Assay

Control and GSTP1 knockdown PDAC cell lines (MIA PaCa-2, PANC-1, and HPAF-II) were seeded in 6-well plates and incubated for 24 h. Cells were synchronized overnight using serum-free medium and harvested by trypsinization, washed, and re-suspended in 70% ethanol overnight at 4 °C. Finally, 70% ethanol was removed, and cells were re-suspended in PBS containing 50 μg/mL propidium iodide (VWR Life Technologies) and 1 μg/mL RNase A (Biotium, Fremont, CA, USA). Flow cytometry was performed using BD Accuri C6 equipment to determine the cell population in each phase of the cell cycle. The data represent the average ± standard deviation of three independent experiments with three technical replicates for each treatment.

### 4.8. Detection of ROS Levels by the 2,7-Dichlorodihydrofluorescein Diacetate (CM-H_2_DCFDA) Assay

Control and GSTP1 knockdown MIA PaCa-2 and HPAF-II cells were resuspended in 20 μM CM-H_2_DCFDA (Life Technologies) in PBS and incubated at 37 °C for 30 min before flow cytometric analysis using a BD Accuri C6. Three technical replicates were included for each experiment, and the experiments were performed in biological triplicates for each cell line. FLOWJO software was used to create histograms. The data represent the average ± standard deviation of three independent experiments with three technical replicates for each treatment.

### 4.9. Orthotopic Tumor Studies

All animal experimental procedures were performed abiding by the protocol approved by North Dakota State University’s Institutional Animal Care and Use Committee (IACUC). Six- to eight-week-old female athymic nude mice (nu/nu) were purchased from The Jackson Laboratory (Bar Harbor, ME, USA). The mice were maintained in sterile conditions using individually ventilated cage (IVC) racks (Allentown and Innovive). The mice were acclimated for 1 week before tumor implantation. PDAC cells were washed twice with PBS, trypsinized, and harvested in serum-containing medium. Harvested cells were washed with serum-free medium and resuspended in PBS. Mice were anesthetized using 3% isoflurane. A small incision was made in the left abdominal flank and control or GSTP1 knockdown cells (7.5 × 10^5^ in 25 μL) were injected into the pancreas using a 27-gauge needle. The abdomen was closed using chromic catgut and ethilon sutures by a 2-layer suture technique. Animals were monitored every day for their food and water intake and for the signs of distress and pain. The tumor volumes were estimated every ten days by abdominal ultrasounds. The mice in the HPAF-II experimental group were euthanized earlier than the previously planned endpoint, as the tumor volumes in the control group were approaching the highest acceptable values as defined in the IACUC protocol. Humane endpoints defined for removing animals from the project were: (1) if/when the tumor burden was estimated to be more than 10–15% of their body weight, if mice demonstrated significant signs of distress or pain, (2) if the tumor interfered with ambulation, if mice exhibited decreased eating or drinking, or (3) if they showed signs of infection [62]. After 4 weeks (HPAF-II group) or 6 weeks (MIA PaCa-2 and PANC-1), animals were euthanized using a CO_2_ chamber (Quietek Model 1, Next Advantage, Troy, NY) that regulates the flow of CO_2_ in the chamber at a rate of 10–30% of the chamber volume per minute. The equipment will not exceed 30% of the chamber volume per minute. These flow rates are compliant with the AVMA regulations for euthanasia of laboratory mice. Animal death was subsequently verified by cervical dislocation. The primary tumor in the pancreata was excised and measured for weight. Each tumor was paraformaldehyde fixed and paraffin embedded for immunohistochemistry. The data represent the average ± standard deviation for the biological replicates.

Ethics approval and consent to participate: All the animal experimental procedures were approved by North Dakota State University’s Institutional Animal Care and Use Committee (protocol number: A17062). The permitted study period on the protocol was from May-2017 to April-2020. North Dakota State University maintains a registration with the United States Department of Agriculture (45-R-002) and an Animal Welfare Assurance with the National Institute of Health-Office of Laboratory Animal Welfare (D16-00156).

### 4.10. Murine Abdominal Ultrasound Imaging

The growth of pancreatic tumors was monitored via abdominal ultrasound imaging every ten days for all animals in the treatment groups (for the HPAF-II group, last ultrasound was performed on D27). A FUJIFILM Vevo3100 ultrasound imaging system (Toronto, ON, Canada) was used to image the pancreata. The animals were anesthetized using 3% isoflurane and were maintained at 2% isoflurane for the course of ultrasound. To support the optimal physiological conditions, mice were kept on the platform maintained at 37 °C. Intraperitoneal administration of 2 mL saline was performed to achieve a higher resolution of abdominal organs. Mice were retained in the supine position and the tumor volumes were calculated using an Mx250 transducer and Vevo Lab Software. The data represent the average ± standard deviation for the biological replicates.

### 4.11. Immunohistochemistry

Tumor tissues were collected and fixed for 24 h in formaldehyde. Paraffin-embedded 5-μm-thick sections of tumor tissues were prepared. Sections were deparaffinized with Histo-Clear and ethanol, followed by antigen retrieval in 10 mM sodium citrate buffer (0.05% Tween 20, pH 6.0) using an autoclave method. The sections were blocked for 20 min in blocking buffer (10% normal goat serum in TBS-T) and incubated with Ki67 (1:100) or cleaved caspase-3 (1:100) overnight at 4 °C. The following day, sections were incubated with CF633-conjugated goat anti-mouse secondary antibody (1:250) for 1 h at room temperature. After mounting a coverslip using Hardset Mounting Medium with 4′,6-diamidino-2-phenylindole (DAPI) (Vector Labs, Burlingame, CA, USA), slides were visualized using a Zeiss inverted Axio Observer Z1 microscope. The percentage of Ki67- or cleaved caspase-3-positive cells was measured based on the number of pink-stained cells relative to the number of blue DAPI-stained nuclei. Immunohistochemistry was performed for all the tumor samples from different treatment groups. The data represent the average ± standard deviation for the biological replicates.

### 4.12. Statistical Analyses

All outcome variables were analyzed using fixed-effects linear models with analysis of variance. For relative GSTP1 expression in different human PDAC cell lines, different cell lines and experimental replicate were the factors. Cell viability was analyzed separately for each PDAC cell line with knockdown line, time, and experimental replicate as the factors. The live and the dead cell population in the scrambled controls of three different cell lines were compared to the same populations in the GSTP1 knockdown cells. The G_0_/G_1_ and G_2_/M populations of scrambled controls were compared to the same populations in GSTP1 knockdown cells. Relative protein expressions of p-JNK, p-ERK, and p-p65 in GSTP1 knockdown cells were analyzed with protein, knockdown line, and experimental replicate as the factors. The relative expression of phosphorylated proteins was compared to total proteins. Relative cleaved caspase-3 expressions were analyzed separately for PDAC cell lines with knockdown lines and experimental replicates as the factors. Pancreas volume was analyzed separately for each PDAC cell line, with knockdown line and day as the factors. Only the results for last ultrasound are presented. The relative tumor weight was analyzed separately for each PDAC cell line using fixed-effects models with knockdown lines as the factor. Welch’s one-way analysis of variance was performed due to the observed heterogeneity of variances. Relative tumor weight, Ki-67-positive cell population, and in vivo cleaved caspase-3 cell population were analyzed separately for each PDAC cell lines using fixed-effects models with knockdown lines as the factor. The Pearson correlation test was done to analyze the association between GSTP1 expression and the survival of patients, post-diagnosis of PDAC.

For any analysis in which an interaction effect was not significant, the interaction effect was dropped from the model for the final analysis. Post-hoc pairwise comparisons using Tukey were performed following significant findings in the overall analysis of variance. All analyses were performed using the MIXED procedure in SAS software version 9.4 (SAS Institute; Cary, NC, USA).

## 5. Conclusions

Currently, pancreatic cancer is the third-leading cause of cancer-related deaths in the US and eighth in the world. PDAC continues to be a major unresolvable health issue at the start of the 21st century. Resistance of PDAC to the conventional treatment approaches has led to an increased interest in identifying promising therapeutic targets. GSTP1 has been associated with tumor promotion and drug resistance in breast, colon, and cervical cancers. Here, we report that GSTP1, a crucial cytoprotective antioxidant protein, plays a critical role in the growth and progression of PDAC cells and tissues. We show that the knockdown of GSTP1 enhances JNK-mediated apoptosis and inhibits NF-κB and ERK-mediated cell survival and proliferation. Our findings are an important first step towards the validation of GSTP1 as a novel therapeutic target to treat pancreatic cancer patients.

## Figures and Tables

**Table 1:** Primer sequences used for measuring mRNA expression via quantitative polymerase chain reaction.

Gene	Forward Sequence	Reverse Sequence
*HPRT*	5′-GAA CGT CTT GCT CGA GAT GTG-3′	5′TCC AGC AGG TCA GCA AAG AAT-3′
*β-Actin*	5′-TTG CCG ACA GGA TGC AGA-3′	5′-GCC GAT CCA CAC GGA GTA CTT-3′
*β-Tubulin*	5′-GTT CGC TCA GGT CCT TTT GG-3′	5′-CCC TCT GTG TAG TGG CCT TTG-3′
*GSTP1*	5′-CAG GAG GGC TCA CTC AAA GC-3′	5′-AGG TGA CGC AGG ATG GTA TTG-3′
*CDKN1A* *HMOX-1*	5′-GGA CAG CAG AGG AAG ACC ATG T-3′ 5′-AAT TCT CTT GGC TGG CTT CCT-3′	5′-GCC GTT TTC GAC CCT GAG A-3′ 5′-CAT AGG CTC CTT CCT CCT TTC C-3′
*Bax*	5′-TTG CTT CAG GGT TTC ATC CA-3′	5′-ACA CTC GCT CAG CTT CTT G-3′
*Bak*	5′-ACA TCA ACC GAC GCT ATG AC-3′	5′-TGG TGG CAA TCT TGG TGA A-3′
*Bcl2*	5′-CGC CCT GTG GAT GAC TGA GTA-3′	5′-CCT CAG CCC AGA CTC ATC A-3′

## Data Availability

All data generated or analyzed during this study are presented in this article. The datasets used and/or analyzed during the current study are available from the corresponding author on reasonable request.

## Supplementary Materials

The following are available online at https://www.mdpi.com/2072-6694/12/6/1501/s1, Figure S1: Unprocessed images for Western blotting results, Figure S2: Clonogenic survival assay results for GSTP1 knockdown MIA PaCa-2 cells.
