# Supplementary material for: Glutathione S-Transferase pi-1 Knockdown Reduces Pancreatic Ductal Adenocarcinoma Growth by Activating Oxidative Stress Response Pathways
Source: Cancers (Basel). 2020 Jun 9;12(6):1501. doi: 10.3390/cancers12061501 (PMC7352757; doi:10.3390/cancers12061501)
Supplement: Supplementary file 1 [file cancers-12-01501-s001.pdf]

Supplementary Materials: Glutathione S-Transferase pi-1 Knockdown Reduces Pancreatic Ductal Adenocarcinoma Growth by Activating Oxidative Stress Response Pathways

Rahul R. Singh, Jiyan Mohammad, Megan Orr and Katie M. Reindl

1A

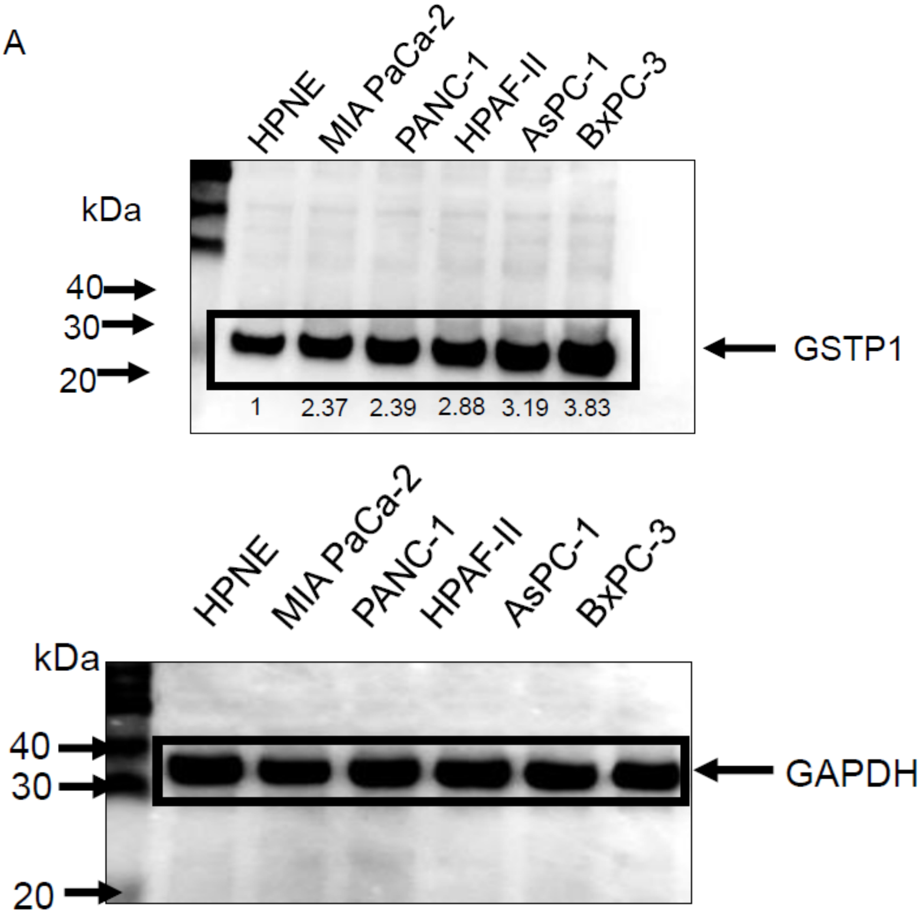

2A

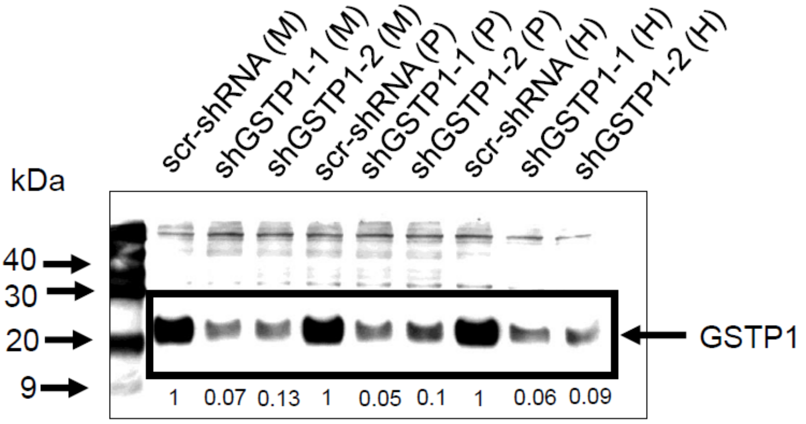

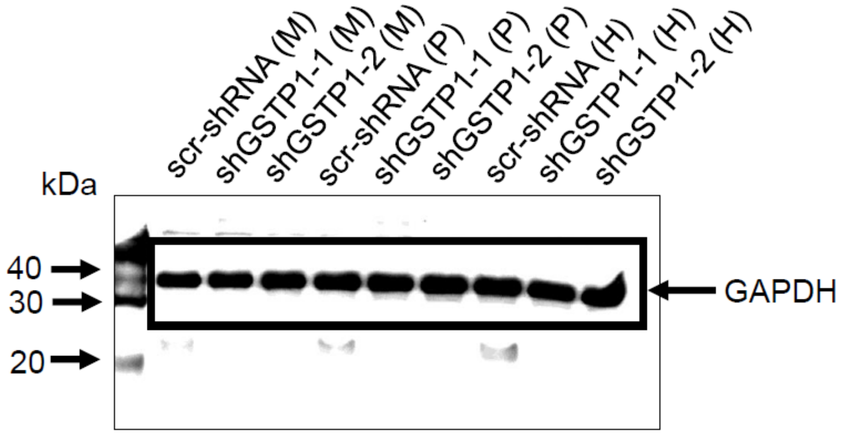
scr-shRNA (M)
shGSTP1-1 (M)
shGSTP1-2 (M)
scr-shRNA (P)
shGSTP1-1 (P)
shGSTP1-2 (P)
scr-shRNA (H)
shGSTP1-1 (H)
shGSTP1-2 (H)
kDa
40
30
20
GAPDH

4A

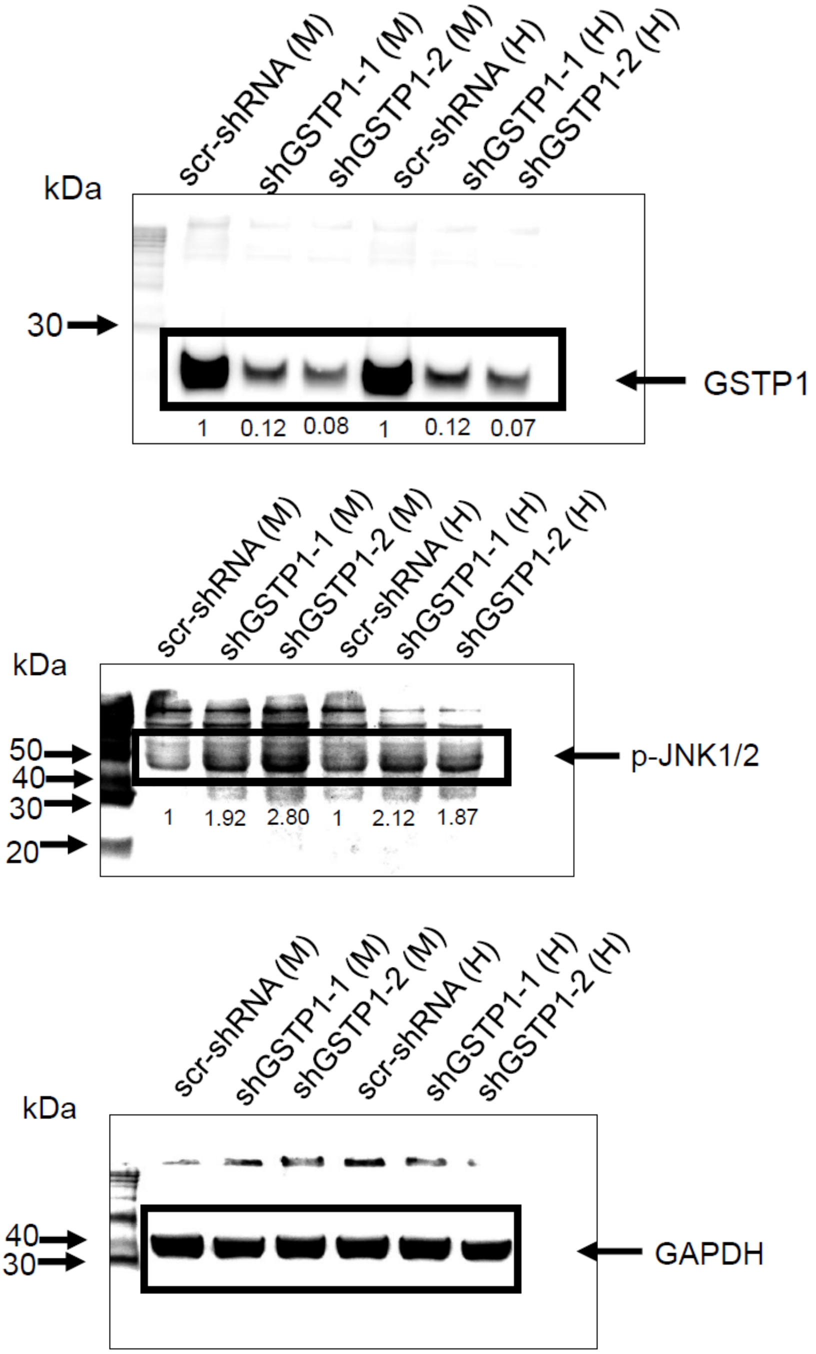
scr-shRNA (M)
shGSTP1-1 (M)
shGSTP1-2 (M)
scr-shRNA (H)
shGSTP1-1 (H)
shGSTP1-2 (H)
kDa
30
GSTP1
1 0.12 0.08 1 0.12 0.07
kDa
50
40
30
20
p-JNK1/2
1 1.92 2.80 1 2.12 1.87
kDa
40
30
GAPDH

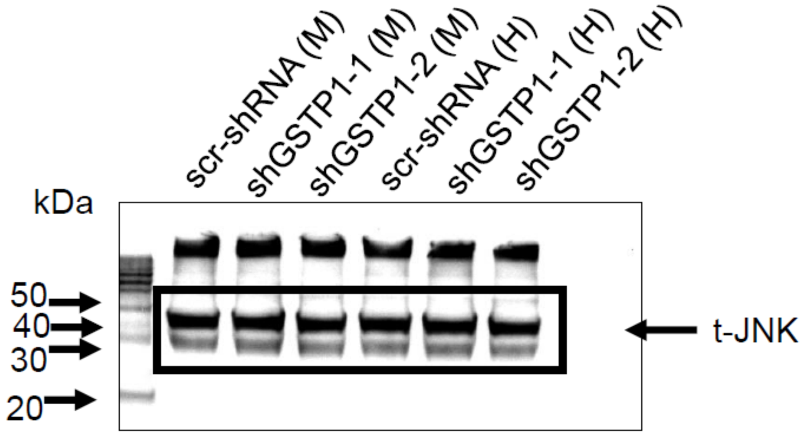
scr-shRNA (M)
shGSTP1-1 (M)
shGSTP1-2 (M)
scr-shRNA (H)
shGSTP1-1 (H)
shGSTP1-2 (H)
kDa
50
40
30
20
t-JNK

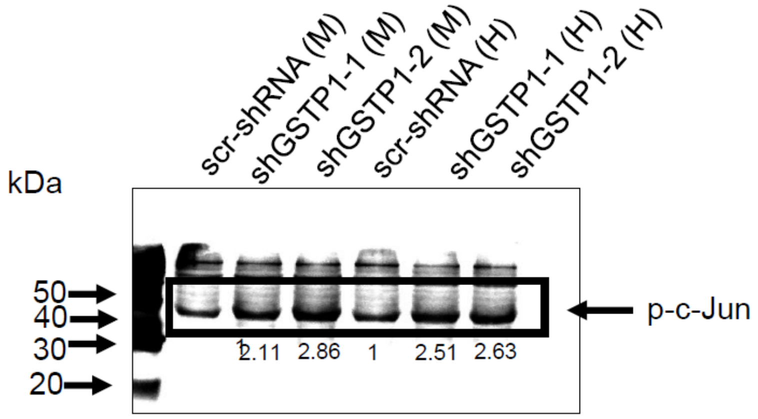
scr-shRNA (M)
shGSTP1-1 (M)
shGSTP1-2 (M)
scr-shRNA (H)
shGSTP1-1 (H)
shGSTP1-2 (H)
kDa
50
40
30
20
p-c-Jun
1 2.11 2.86 1 2.51 2.63

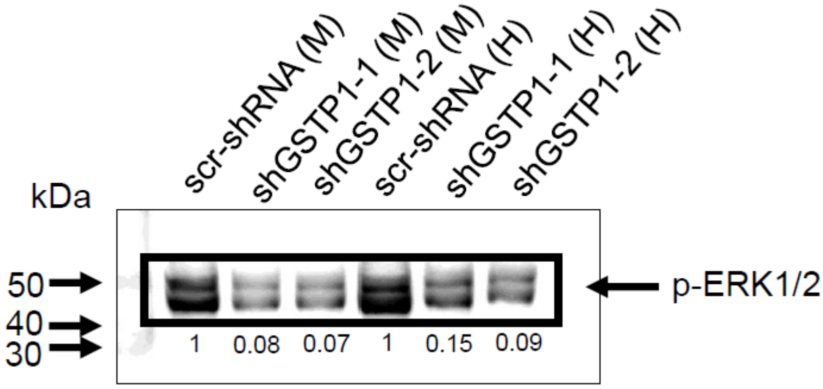
scr-shRNA (M)
shGSTP1-1 (M)
shGSTP1-2 (M)
scr-shRNA (H)
shGSTP1-1 (H)
shGSTP1-2 (H)
kDa
50
40
30
p-ERK1/2
1 0.08 0.07 1 0.15 0.09

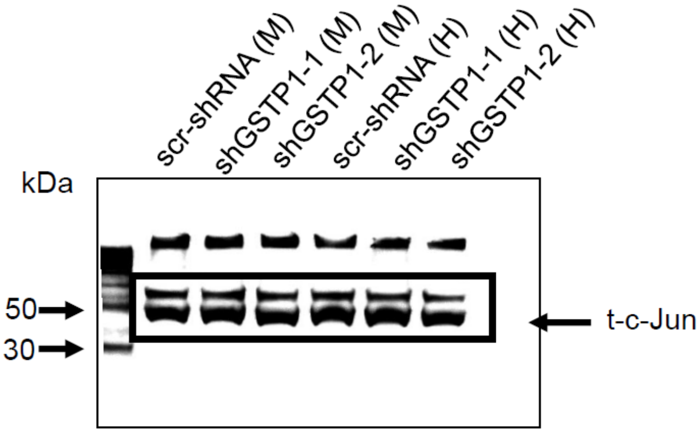
scr-shRNA (M)
shGSTP1-1 (M)
shGSTP1-2 (M)
scr-shRNA (H)
shGSTP1-1 (H)
shGSTP1-2 (H)
kDa
50
30
t-c-Jun

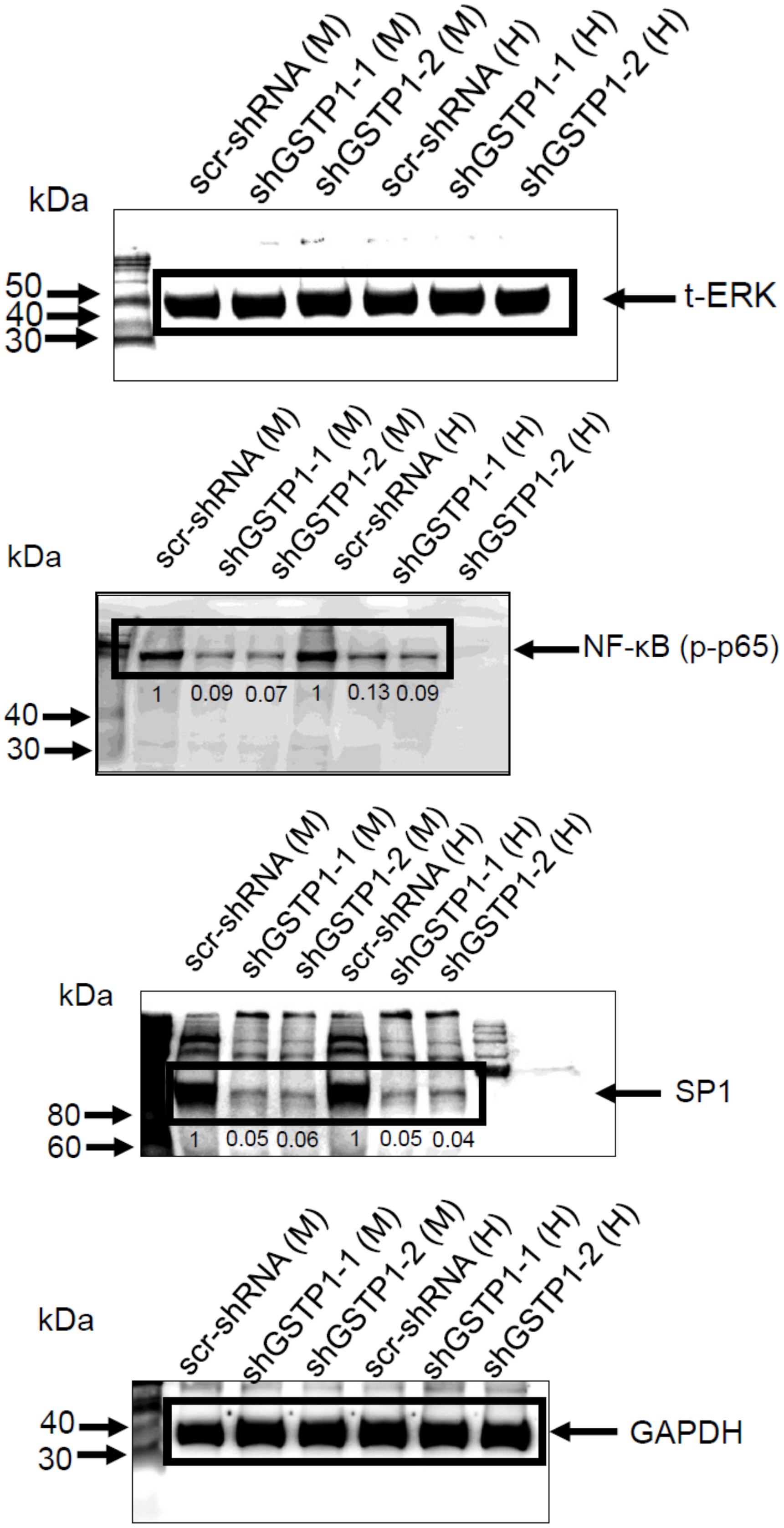
scr-shRNA (M)
shGSTP1-1 (M)
shGSTP1-2 (M)
scr-shRNA (H)
shGSTP1-1 (H)
shGSTP1-2 (H)
kDa
50
40
30
t-ERK
scr-shRNA (M)
shGSTP1-1 (M)
shGSTP1-2 (M)
scr-shRNA (H)
shGSTP1-1 (H)
shGSTP1-2 (H)
kDa
NF-κB (p-p65)
1 0.09 0.07 1 0.13 0.09
40
30
scr-shRNA (M)
shGSTP1-1 (M)
shGSTP1-2 (M)
scr-shRNA (H)
shGSTP1-1 (H)
shGSTP1-2 (H)
kDa
SP1
80
60
1 0.05 0.06 1 0.05 0.04
scr-shRNA (M)
shGSTP1-1 (M)
shGSTP1-2 (M)
scr-shRNA (H)
shGSTP1-1 (H)
shGSTP1-2 (H)
kDa
40
30
GAPDH

4D

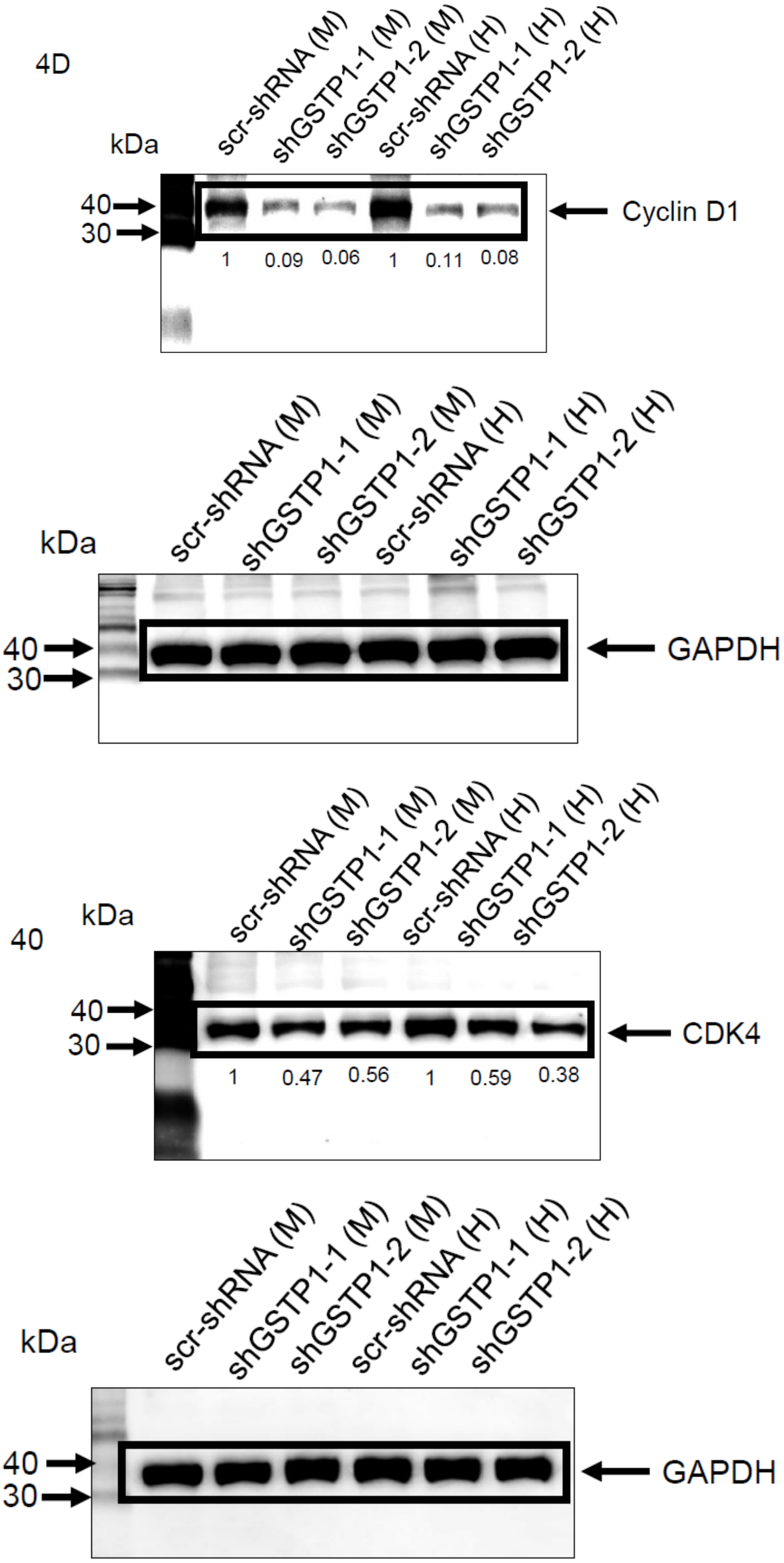
scr-shRNA (M)
shGSTP1-1 (M)
shGSTP1-2 (M)
scr-shRNA (H)
shGSTP1-1 (H)
shGSTP1-2 (H)
kDa
40
30
Cyclin D1
1
0.09
0.06
1
0.11
0.08
scr-shRNA (M)
shGSTP1-1 (M)
shGSTP1-2 (M)
scr-shRNA (H)
shGSTP1-1 (H)
shGSTP1-2 (H)
kDa
40
30
GAPDH
scr-shRNA (M)
shGSTP1-1 (M)
shGSTP1-2 (M)
scr-shRNA (H)
shGSTP1-1 (H)
shGSTP1-2 (H)
40
kDa
40
30
CDK4
1
0.47
0.56
1
0.59
0.38
scr-shRNA (M)
shGSTP1-1 (M)
shGSTP1-2 (M)
scr-shRNA (H)
shGSTP1-1 (H)
shGSTP1-2 (H)
kDa
40
30
GAPDH

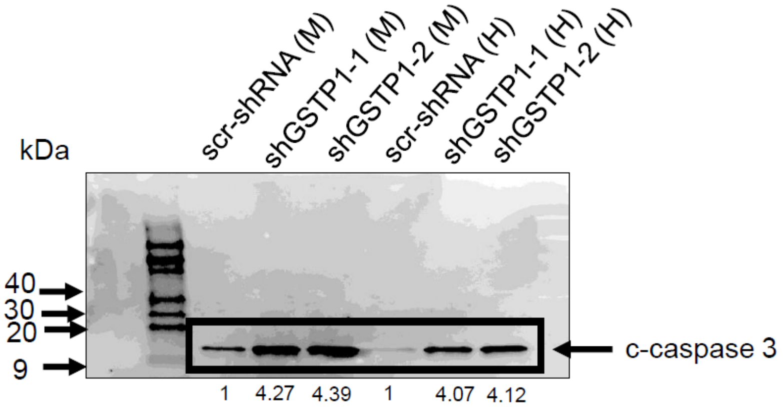
scr-shRNA (M)
shGSTP1-1 (M)
shGSTP1-2 (M)
scr-shRNA (H)
shGSTP1-1 (H)
shGSTP1-2 (H)
kDa
40
30
20
9
c-caspase 3
1 4.27 4.39 1 4.07 4.12

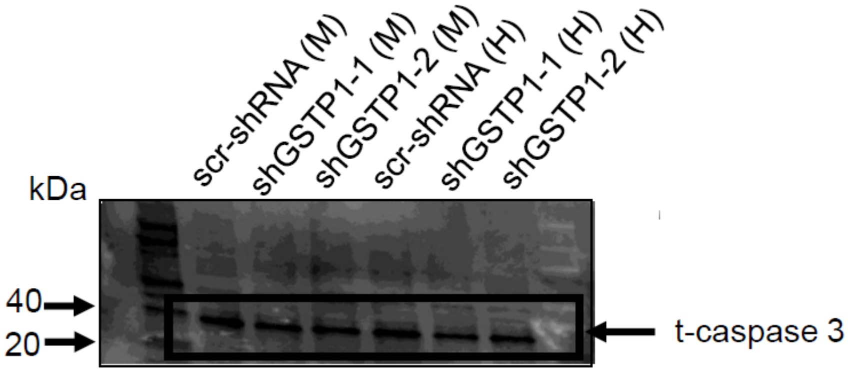
scr-shRNA (M)
shGSTP1-1 (M)
shGSTP1-2 (M)
scr-shRNA (H)
shGSTP1-1 (H)
shGSTP1-2 (H)
kDa
40
20
t-caspase 3

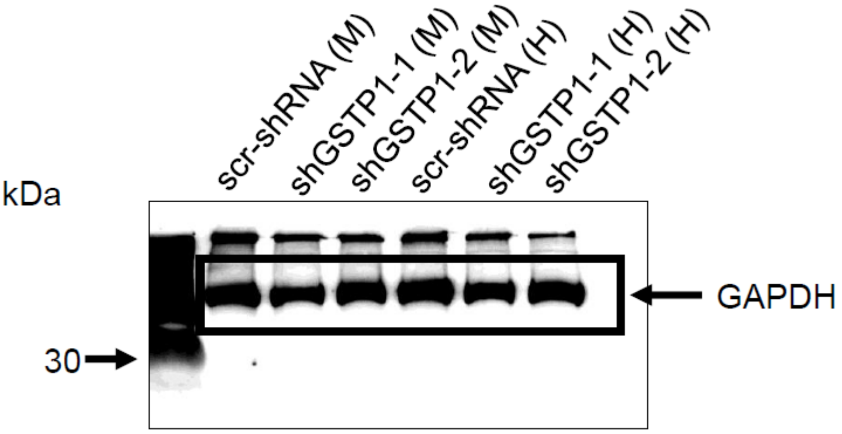
scr-shRNA (M)
shGSTP1-1 (M)
shGSTP1-2 (M)
scr-shRNA (H)
shGSTP1-1 (H)
shGSTP1-2 (H)
kDa
30
GAPDH

5B

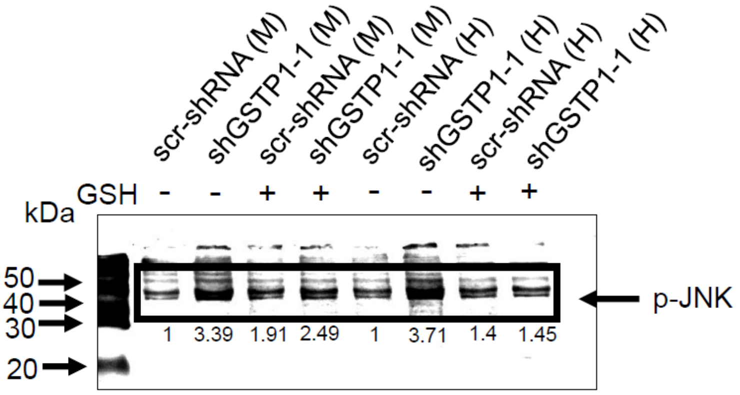
scr-shRNA (M)
shGSTP1-1 (M)
scr-shRNA (M)
shGSTP1-1 (M)
scr-shRNA (H)
shGSTP1-1 (H)
scr-shRNA (H)
shGSTP1-1 (H)
GSH - - + + - - + +
kDa
50
40
30
20
p-JNK
1 3.39 1.91 2.49 1 3.71 1.4 1.45

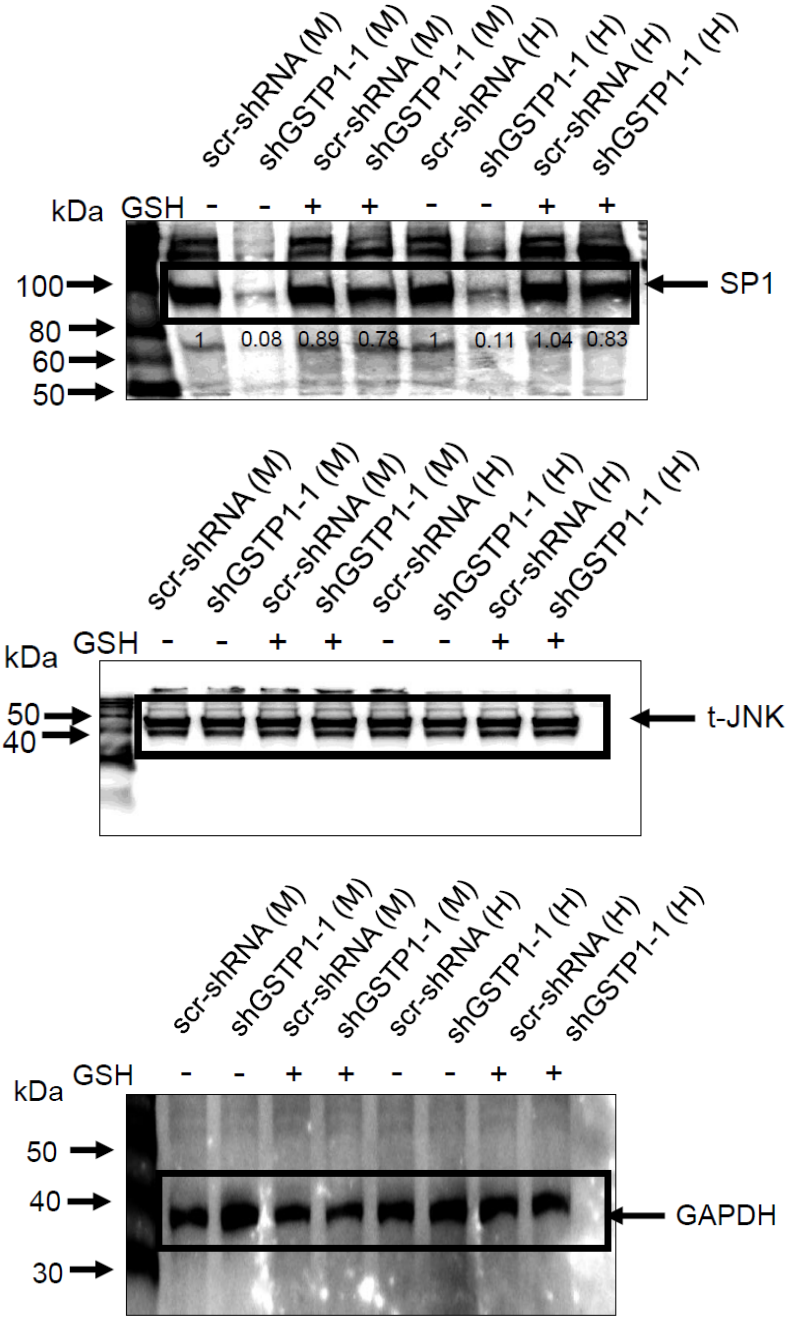

Figure S1. Unprocessed images for Western blotting results. Densitometry values are shown in the blot images. (M): MIA PaCa-2, (P): PANC-1 and (H): HPAF-II.

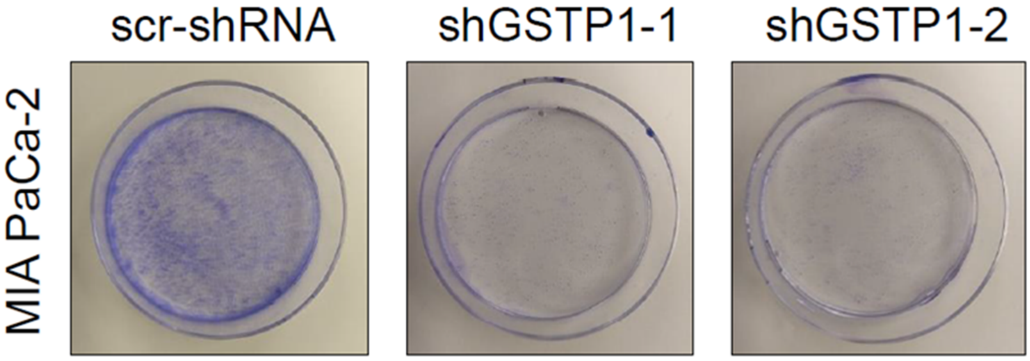

Figure S2. Clonogenic survival experiment results obtained for control (scr-shRNA) and GSTP 1 knockdown (shGSTP 1-1 and GSTP 1-2) MIA PaCa-2 cells.

© 2020 by the authors. Submitted for possible open access publication under the terms and conditions of the Creative Commons Attribution (CC BY) license (http://creativecommons.org/licenses/by/4.0/).
